# Hand-Foot Syndrome as a Rare Side Effect of Bleomycin

**DOI:** 10.12669/pjms.42.7.12343

**Published:** 2026-07

**Authors:** Aateqa Hashmi, Maryam Abid, Amjad Zafar, Muhammad Abbas Khokhar

**Affiliations:** 1 Aateqa Hashmi, MBBS, King Edward Medical University, Mayo Hospital, Lahore, Pakistan; 2 Maryam Abid, BSc, MBBS, MD, SCE Medical Oncology Department of Oncology and Radiation Therapy, King Edward Medical University, Mayo Hospital, Lahore, Pakistan; 3 Amjad Zafar, MBBS, FCPS Department of Oncology and Radiation Therapy, King Edward Medical University, Mayo Hospital, Lahore, Pakistan; 4 Muhammad Abbas Khokhar, MBBS, FCPS, MD (USA), DHHM Department of Oncology and Radiation Therapy, King Edward Medical University, Mayo Hospital, Lahore, Pakistan

**Keywords:** Acral erythema, Bleomycin-etoposide-cisplatin (BEP), Chemotherapy, Drug toxicity, Hand Foot Syndrome, modified-BEP (mBEP), Skin desquamation

## Abstract

Hand-Foot Syndrome is a rare adverse reaction to bleomycin-etoposide-cisplatin chemotherapy, characterized by acral skin desquamation, hyperpigmentation, and swelling involving the hands and feet. We report the case of a 32 years old Asian female, diagnosed with right-sided Stage-IV dysgerminoma, who developed cutaneous symptoms following the second cycle of chemotherapy. She presented with hyperpigmentation, swelling, and desquamation affecting both hands and feet. After consultation with the dermatology department, she was diagnosed with chemotherapy-induced skin toxicity. Bleomycin, along with other cytotoxic agents, was discontinued. Initiation of topical betamethasone therapy resulted in significant clinical improvement over the following weeks. One month after starting the treatment, her feet had healed completely, while her hands continued to improve in the recovery phase with ongoing supportive care. This case highlights a rare dermatologic complication of bleomycin-etoposide-cisplatin therapy, emphasizing the need for further exploration into its prevention and effective treatment strategies.

***List of Abbreviations:***

**BEP:** Bleomycin-etoposide-cisplatin, **HFS:** Hand-foot syndrome, **mBEP:** modified-BEP.

## INTRODUCTION

Bleomycin is an antitumor antibiotic first isolated in 1966 from *Streptomyces verticillus*.^1^ It has since demonstrated efficacy in the oncologic management of various malignancies, most notably Hodgkin’s lymphoma and germ cell tumors. Its toxicity profile includes pulmonary and dermatologic adverse effects, the former being more prevalent owing to the presence of the inactivating enzyme hydrolase in lower concentrations in the lungs and skin compared to other tissues.^2^,^3^

Cutaneous toxicity associated with bleomycin includes nail bed changes, hyperkeratosis, flagellate skin rash, skin peeling, and, in rare cases, palmar-plantar desquamation. Knowledge and early recognition of such uncommon adverse events are essential for timely intervention, allowing for personalized modification of chemotherapeutic regimens and prevention of further toxicity.^4^

We herein present the case of a young female who developed palmar-plantar desquamation following the second cycle of bleomycin-etoposide-cisplatin (BEP) chemotherapy for her Stage-IV dysgerminoma.

## CASE PRESENTATION

**Fig.1:**
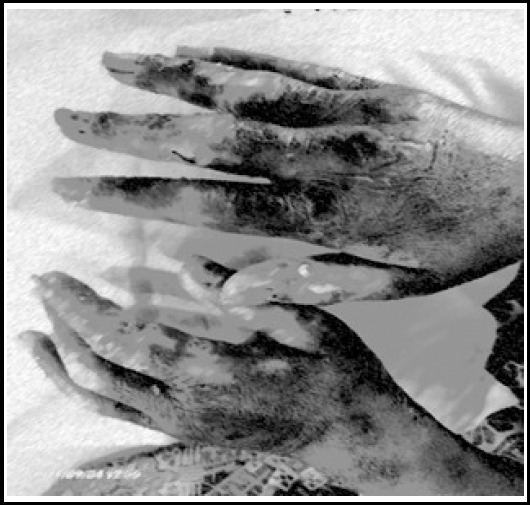
Desquamation and hyperpigmentation of hands.

**Fig.2:**
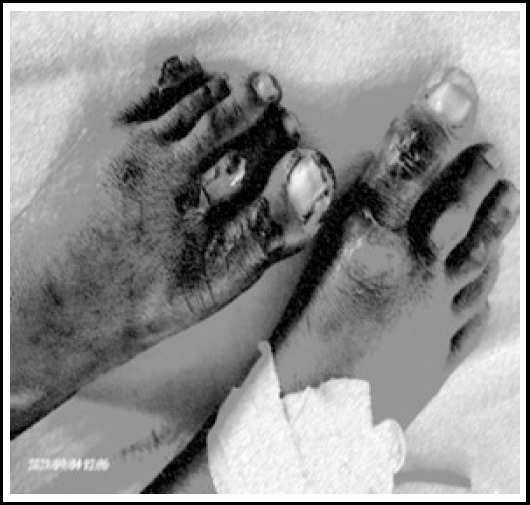
Desquamation and hyperpigmentation of feet.

A 32 years old Asian female, recently diagnosed with Stage-IV right-sided dysgerminoma, underwent right salpingo-oophorectomy and excision of a pelvic mass, without nodal dissection. She was started on a planned three-cycle regimen of BEP chemotherapy. Two weeks after her second cycle (bleomycin 30 units D1, D8, D15; cisplatin 30 mg D1-5; etoposide 140 mg D1-5), the patient presented with desquamation of both hands and feet, accompanied by hyperpigmentation and swelling (Fig.1 and 2). She had no significant past medical, surgical, allergic, or family history.

On physical examination, the patient’s extremities were warm and non-tender. In addition to cutaneous manifestations, she developed oral mucositis, angular stomatitis, pharyngitis, and dehydration likely due to chemotherapy-induced vomiting. Laboratory workup revealed pancytopenia, elevated ALP, and neutrophilic leukocytosis, hinting at an acute reaction in her body. Arterial Doppler studies of both arms were unremarkable.

A clinical diagnosis of chemotherapy-induced skin desquamation was made. After inpatient admission, the dermatology team was consulted, leading to discontinuation of chemotherapy along with initiation of treatment with topical betamethasone. Oral candidiasis was managed with triamcinolone (Kenalog). During admission, she developed acute kidney injury and low-grade intermittent fever, suspected to be related to cisplatin nephrotoxicity, though no urinary symptoms were reported. One month later, the patient’s feet healed completely, and her hands were in a healing phase with significant improvement. The patient received psychiatric support and culturally sensitive counseling to help maintain emotional well-being during cancer treatment and adverse side effects.

## DISCUSSION

Hand-foot syndrome (HFS), or palmar-plantar erythrodysesthesia, is a common adverse effect of chemotherapeutic agents such as doxorubicin, cytarabine, cisplatin, and 5-fluorouracil. Though rarely, it has also been reported with bleomycin therapy.^5^ The condition typically begins with tingling sensations in the palms and/or soles, followed by painful erythema, and may culminate in desquamation and ulceration.

A previously reported case described the emergence of acral erythema following an increase in bleomycin dose, manifesting as round, painful, and edematous lesions affecting both palmar and plantar surfaces. Symptoms resolved following cessation of chemotherapy and treatment with topical betamethasone and oral antihistamines.^2^ Our patient exhibited similar cutaneous toxicity but with additional features, including plantar-palmar desquamation and mucocutaneous gastrointestinal side effects.

Although a biopsy of the acral lesions would have provided histopathological confirmation, resource limitations in this setting precluded tissue sampling; nevertheless, acknowledging the diagnostic value of biopsy strengthens the completeness and transparency of our report.

Given its potential for pulmonary and dermatologic toxicity, the use of bleomycin has declined in recent years.^6^ Modified regimens such as etoposide and cisplatin (EP) without bleomycin have demonstrated comparable efficacy with a more favorable toxicity profile and lower cost.^7^ Similarly, a modified BEP (mBEP) regimen has shown promise in germ cell tumor management, delivering effective oncological outcomes while minimizing pulmonary toxicity.

Emerging pharmacogenetic strategies offer further precision in chemotherapeutic planning. One compelling study suggests that genetic polymorphisms affecting drug-metabolizing enzymes may determine bleomycin toxicity risk. Individuals classified as “slow metabolizers” may accumulate higher systemic concentrations, increasing the likelihood of adverse effects, whereas “fast metabolizers” may tolerate standard dosages better.^8^

Several adjunctive strategies have also been explored for mitigating HFS symptoms. Application of cold packs to the wrists and ankles during chemotherapy may reduce acral blood flow and thereby limit local drug accumulation and inflammation.^9^ Pharmacologic interventions such as oral celecoxib, a COX-2 selective inhibitor, have demonstrated efficacy in reducing moderate to severe HFS.^10^ Curcumin, the active ingredient of turmeric, has come to attention due to preliminary evidence suggesting its role in both treatment and prevention of chemotherapy-induced adverse effects without compromising therapeutic efficacy. Pyridoxine (vitamin B6) has also been studied as a protective agent against chemotherapy-induced skin toxicity; however, results remain inconsistent and warrant further clinical validation.^10^

Bleomycin is the most likely causative agent responsible for this patient’s hand-foot syndrome, given its well-recognized and distinctive dermatologic toxicity profile. A key factor supporting this association is the relatively low activity of bleomycin hydrolase in the skin, which results in impaired drug inactivation and subsequent local accumulation within cutaneous tissues. This pharmacologic characteristic renders the skin particularly vulnerable to bleomycin-induced toxicity. Unlike cisplatin and etoposide, which rarely induce acral desquamation, bleomycin has documented associations with palmar-plantar reactions that closely resemble this presentation. The temporal relationship between symptom onset and cumulative exposure to bleomycin further strengthens this attribution, as bleomycin-related skin toxicity is often dose dependent and may manifest after repeated cycles of therapy. Additionally, prior reports describing bleomycin-induced palmar-plantar reactions lend further support to its role as the offending agent in this scenario. Moreover, evidence that etoposide and cisplatin (EP) regimens without bleomycin achieve comparable efficacy with fewer cutaneous adverse effects reinforces bleomycin as the most plausible offending agent.

Given the psychological toll of disfiguring side effects, our patient received psychiatric support and culturally sensitive counseling, which she reported as profoundly helpful in maintaining emotional resilience during the treatment. This case underscores the necessity of a holistic, multidisciplinary approach, uniting oncology, dermatology, psychiatry, and radiology, to optimize outcomes for complex cancer patients. Although bleomycin-induced skin toxicity is rare, similar presentations from other agents make an accurate diagnosis crucial. As personalized medicine evolves, integrating genetic insights and supportive therapies may redefine chemotherapy, not just as a battle against disease, but as a journey of healing, dignity, and hope.

## References

[1] Verma SP, Subbiah A, Kolar Vishwanath V, Dutta TK (2016). Bleomycin-induced skin toxicity: Is it always flagellate erythema?. BMJ Case Rep.

[2] Tsuboi H, Yonemoto K, Katsuoka K (2005). A case of bleomycin-induced acral erythema with eccrine squamous syringometaplasia and summary of reports of AE with ESS in the literature. J Dermatol.

[3] Lazo JS, Humphreys CJ (1983). Lack of metabolism as the biochemical basis of bleomycin-induced pulmonary toxicity. Proc Natl Acad Sci USA.

[4] Agrawal C, Talwar V, Saini R, Babu P (2017). Flagellate rash: an unusual complication of bleomycin therapy - a case report with brief review of literature. Indian J Med Paediatr Oncol.

[5] Baack BR, Burgdorf WH (1991). Chemotherapy-induced acral erythema. J Am Acad Dermatol.

[6] Biswas A, Julka PK (2016). Bleomycin-induced flagellate erythema in a patient with thalamic mixed germ cell tumour: report of a rare adverse effect. J Egypt Natl Cancer Inst.

[7] McHugh DJ, Funt SA, Silber D, Knezevic A, Patil S, O'Donnell D (2020). Adjuvant chemotherapy with etoposide plus cisplatin for patients with pathologic stage II nonseminomatous germ cell tumors. J Clin Oncol.

[8] Del Re M, Latiano T, Fidilio L, Restante G, Morelli F, Maiello E (2017). Unusual gastrointestinal and cutaneous toxicities by bleomycin, etoposide, and cisplatin: a case report with pharmacogenetic analysis to personalize treatment. EPMA J.

[9] Zimmerman GC, Keeling JH, Lowry M, Medina J, Von Hoff DD, Burris HA (1994). Prevention of docetaxel-induced erythrodysesthesia with local hypothermia. J Natl Cancer Inst.

[10] Macedo LT, Lima JPN, dos Santos LV, Sasse AD (2014). Prevention strategies for chemotherapy-induced hand-foot syndrome: a systematic review and meta-analysis of prospective randomised trials. Support Care Cancer.

